# Work-family enrichment: A potential buffer of inflammation among black adults?

**DOI:** 10.1016/j.bbih.2022.100517

**Published:** 2022-09-19

**Authors:** Nicholas D. Thomas, Shannon C. Montgomery, Benjamin Behers, Eduardo Reyes, Thomas Ledermann, Joseph G. Grzywacz

**Affiliations:** a Florida State University College of Medicine, FL, USA; b Florida State University College of Health and Human Sciences, FL, USA

**Keywords:** MIDUS, Work-family, Enrichment, Inflammation, Interleukin-6, CVD, Black Americans

## Abstract

**Background:**

Inflammation plays a known role in the development of cardiovascular disease (CVD), the leading cause of death in the United States and a condition that disproportionately affects Blacks. Although social stressors are frequently studied, the role of positive experiences in inflammation and its potential for CVD remains understudied. To address this gap, we examined the relationship between work family enrichment and inflammation in a population-based sample of working adults.

**Methods:**

Participants were 447 working adults from Refresher Cohort of the National Study of Midlife Development in the United States (MIDUS) and the oversample of Blacks from the Milwaukee, WI. Serum concentration of pro-inflammatory biomarkers (IL-6/sIL-6r; CPR; Fibrinogen) were obtained via blood draw. Family-to-work enrichment (FtoWE) and work-to-family enrichment (WtoFE) were each assessed with four established survey questions.

**Results:**

Blacks had higher concentrations of IL-6, CRP and Fibrinogen, and lower levels of sIL-6r than whites. A significant inverse relationship was observed between WtoFE and systemic inflammation as well as WtoFE and serum IL-6 concentration.

**Conclusions:**

Individuals who perceived a stronger enhancing effect from work onto family showed lower levels of systemic inflammation and decreased concentrations of the pro-inflammatory cytokine IL-6; highlighting the potential work-family enrichment or other positive experiences may have in buffering the negative cardiovascular effects of inflammation. However, variation between racial groups remain undetermined.

## Highlights

• Higher concentrations of pro-inflammatory biomarkers were seen among Blacks.
• Greater WFE conferred less systemic inflammation and lower concentrations of IL-6.
• Positive experiences from work to family may buffer inflammation, improving CVD risk.

## 1 Introduction

Inflammation is increasingly implicated in the development of cardiovascular disease (CVD) – the leading cause of death in the United States (US) (Graham, 2015). From 1999 to 2017, CVD death rates have dropped significantly for all age, race, and gender groups; however, the Black population still faces the highest rates of CVD mortality and morbidity (Curtin, 2019). The increased burden of psychosocial stressors such as low income and family strain faced by Blacks has been linked to earlier progression of CVD and other chronic illnesses such as obesity and diabetes (Friedman and Herd, 2010; Gebreab et al., 2017; Merker et al., 2022; Rohleder, 2014; Suglia et al., 2020).

Previous studies found higher levels of clinical inflammatory markers of CVD risk such as interleukin 6 (IL-6), C reactive protein (CRP), and fibrinogen in blood samples of individuals with chronically increased stressors (Feghali et al., 1997; Kiecolt-Glaser et al., 2020; Lagraauw et al., 2015; Puzianowska-Kuźnicka et al., 2016; Rohleder, 2014). These biomarkers play specific roles in the inflammatory process, contributing to both initiation and progression of CVD (Jordan et al., 2017; Rossi et al., 2015). However, with IL-6's central role in inflammation receiving greater attention, a more in-depth investigation is warranted. In clinical studies, inhibition of the IL-6 signaling has demonstrated great success in combatting inflammation (Rossi et al., 2015; Smolen et al., 2013; Verma et al., 2002). Furthermore, the soluble IL-6 receptor (sIL-6r) has become a therapeutic target for CVD, with studies suggesting that sIL-6r may play a critical mediating role between IL-6 and CVD. Specifically, evidence suggests that pharmacologically blocking sIL-6r attenuates the heart's pro-inflammatory response to IL-6 (Casas and Hingorani, 2012; Jordan et al., 2017).

The potential for positive experiences to offer insight on racial variation in inflammation has largely been overlooked as researchers have mainly focused on the pathophysiology between stressor related inflammation and CVD. Sin et al. (2015), drawing on a large general population sample of adults, reported that more frequent exposure to positive experiences was associated with reduced serum IL-6 concentrations. Further, they found that more frequent exposure to positive experiences was associated with reduced serum fibrinogen among women. This same study also notes that Blacks report fewer positive daily experiences and had elevated IL-6, CRP and Fibrinogen concentrations in comparison to Whites (N. L. Sin et al., 2015).

Success in the core domains of work and family life is a critical area to consider when exploring psychosocial determinants of health (Grzywacz and Marks, 2000; Liu et al., 2022). Work-family enrichment is a concept that captures positive experiences resulting from synergy between employed adults’ “work” and “family lives” (Carlson et al., 2006, 2010; Greenhaus and Powell, 2006; Grzywacz and Marks, 2000). The synergies between work and family can follow two distinct directions, that is from family-to-work (FtoWE) as when skills learned as a parent may be used to manage situations at work, from work-to-family (WtoFE), like when an achievement at work (i.e. acquiring a new skill, completing a goal) elevates mood or sense of self and results in positivity at home. Evidence suggests the directions of work-family enrichment are distinct (Grzywacz and Butler, 2005), and results from meta-analysis suggest both WtoFE and FtoWE have a positive relationship with physical and mental health (McNall et al., 2010).

Several strands of evidence suggest the directions of work-family inflammation may be related with inflammation. Meta-analytic results indicate that generalized work-family enrichment is associated with less burnout (Zhang et al., 2018), a psychological state strongly linked with inflammation (Bayes et al., 2021). Both WtoFE and FtoWE have been associated with decreased risk of depression and anxiety disorder (Grzywacz and Bass, 2003), which have recursive associations with inflammation (Kiecolt-Glaser et al., 2015). Previous studies have also implicated personality with work-family enrichment, showing that greater extraversion and openness to experiences were associated with greater WtoFE and FtoWE (Michel et al., 2011). Similarly, individuals with higher positive affectivity had higher levels of both WtoFE and FtoWE (Michel and Clark, 2009; Tement and Korunka, 2013). However, self-reported levels of WtoFE and FtoWE are remarkably stable across 30 years and not attributed to personality traits or well-being (Kim et al., 2021). Instead, Kim and colleagues contend that work-family enrichment is a positive experience that is actively constructed and sustained, thereby creating enduring potential for well-being. Collectively, these threads of evidence suggest that WtoFE and FtoWE may provide protective buffer against inflammation, in turn lowering CVD risk.

The goal of this study was to improve understanding of the potential role of WtoFE and FtoWE in CVD and racial inequalities in CVD by studying its association with inflammation. The study goal was pursued through three primary aims designed to: (1) document variation in selected indicators of inflammation by race among working adults; (2) determine if WtoFE and FtoWE differs between White and Black individuals in a community-based sample of US adults; and (3) investigate the relationship between WtoFE and FtoWE and systemic inflammation, as well as inflammation as a function of IL-6/sIL-6 pathway. We hypothesize that higher concentrations of inflammatory biomarkers and lower levels of WtoFE and FtoWE will be observed in the Black cohort, and WtoFE and FtoWE will be inversely associated with inflammation.

## 2 Methods

### 2.1 Sample characteristics

Data for this study were obtained from a nationally representative sample of adults who participated in the Midlife in the United States (MIDUS) Refresher and Refresher Milwaukee Projects. The original MIDUS I project was a national longitudinal study that aimed to investigate psychological and social factors that may account for age-related variations in health. In 2011–2014, the MIDUS Refresher study replenished the original MIDUS I cohort. Survey data was collected on demographic, physical and mental health information via a 30-min phone interview followed by two 50-page mailed self-administered questionnaires (SAQ). From 2012 to 2013, the MIDUS Milwaukee Refresher study recruited an over-sample of Black adults residing in Milwaukee, WI, aged 25 to 64. Milwaukee respondents were interviewed in their homes using a 2.5-h Computer Assisted Personal Interview (CAPI) protocol and afterwards completed a SAQ. Data on demographics and work-family enrichment were obtained via self-report.

The MIDUS Refresher Projects also included a biomarker component wherein data collection spanned 2012–2016. Specimens collected included a fasting blood draw, 12-h urine, and saliva sample. Protocol for this project included assessments by clinicians or trained staff. Day 1 consisted of a physical exam and a medical history, and blood samples were taken after fasting on day 2. Biomarker levels reflect metabolic functioning among participants. For the current study, the analytic sample was limited to Whites and Blacks in the MIDUS Refresher and Blacks from the Milwaukee Refresher who completed the SAQ and participated in the biomarker component of the project (N = 447). Due to small group sizes, Blacks in the MIDUS Refresher and Milwaukee Refresher samples were combined into one sample (MIDUS Refresher and Milwaukee Refresher Black; N = 82) and MIDUS White (N = 365) were analyzed as separate groups.

## 3 Measures

### 3.1 Work-family enrichment

Bidirectional work-family enrichment was assessed using the self-administered questionnaire items developed in the original MIDUS study (Grzywacz and Marks, 2000). WtoFE was assessed using four questions pertaining to the advantageous effects at home due to positive work experiences such as, “Do the things you do at work help you deal with personal and practical issues at home?” and “Does having a good day on your job make you a better companion when you get home?” FtoWE was determined via four items probing beneficial effects of family life for the work (e.g., “Does your home life help you relax and feel ready for the next day's work?” and “Does talking with someone at home help you deal with problems at work?“). All items used to measure WtoFE and FtoWE were placed on a 5-point Likert scale and reverse-coded so that higher scores reflected higher levels of the assessed construct. The Cronbach's alpha was 0.716 for WtoFE and 0.666 for FtoWE.

### 3.2 Biomarkers of inflammation

Four pro-inflammatory indicators were examined (sIL-6r, IL-6, CRP, and Fibrinogen). Venous blood samples were collected from participants following overnight fasting. Samples were stored in a −65 °C freezer until assayed. sIL-6r, IL-6, were assayed at the MIDUS Biocore Lab using a V-plex Custom human Cytokine Kit (Meso Scale Diagnostics, Rockville, MD). Inter-assay coefficients of variation were sIL-6r (5.3%), IL-6 (15.7%), and intra-assay coefficients of variation were sIL-6r (1.3%), IL-6 (4.7%). C-Reactive Protein (CRP) and Fibrinogen were assayed at the Laboratory for Clinical Biochemistry Research (University of Vermont, Burlington, VT) and measured using a BN II nephelometer with a particle enhanced immunonephelometric assay. Intra-assay and inter-assay coefficients of variation for CRP were 1.08–4.3% and 2.3–4.4% and for Fibrinogen were 2.6% and 2.7% respectively. Serum concentration of biomarkers were used to determine *in vivo* levels of inflammation. In line with previous research (Friedman et al., 2015) distributions for both IL-6 and CRP were positive skewed, so they were log-transformed for statistical analyses. Fibrinogen was scaled by a factor of 100 to make values comparable with the other measures (necessary for model estimation). IL-6, CRP, and Fibrinogen data were available for 442 participants included in this study.

### 3.3 Covariates

Respondents reported their highest level of education on a continuous scale from 6th grade or less to graduate school, doctorate, or advanced professional degree. Highest level of education completed was dichotomized into high school education or below, or some college education or above. Body mass index was measured by study staff and was computed by dividing weight (in kilograms) by height squared (in meters). Height measure (in centimeters) was multiplied divided by 100 to get the height in meters. Participants were dichotomized as obese or not obese based on their Body Mass Index (BMI), with those with a BMI of 30 or greater coded as one for “obese,” zero or “not obese” otherwise. The number of chronic conditions in the past 12 months was measured by a self-reported continuous scale. Based on the upper quartile range, participants who had 4 or more chronic conditions were dichotomized as having co-morbid chronic conditions, whereas 3 or fewer chronic conditions was dichotomized as 0 co-morbid chronic conditions. Perceived stress was measured with the perceived stress scale (Cohen et al., 1983). Responses were scored on a Likert scale (0–4), with positive items reverse-coded so that higher values reflected greater perceived stress, and then summed for a range of 0–40 (Cronbach's alpha 0.859). Pre-tax income was included as a continuous variable, measured as pay from all jobs and income from other sources such as retirement, unemployment insurance, food stamps, and gifts from family and friends. Positive affect was a constructed variable measured from 6 items (i.e., during that last 30 days did you feel cheerful, in good spirits …) with responses ranging from 1 (“all of the time”) to 5 (“none of the time”) that were recoded so that larger values indicate more positive affect and summed (ɑ = 0.931). Extraversion was a constructed variable measured with 5 items asking how well a characteristic (e.g., outgoing, friendly) described the participant on a scale of 1 (‘a lot”) to 4 (“not at all”) that were recoded so that larger values indicated more extraversion and then averaged (ɑ = 0.754).

### 3.4 Structural equation model

**Fig. 1:**
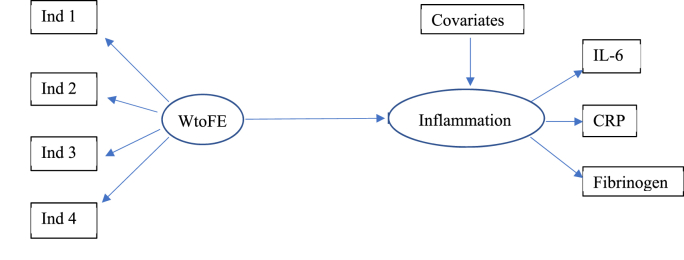
Multiple-group structural equation model.

**Fig. 2:**
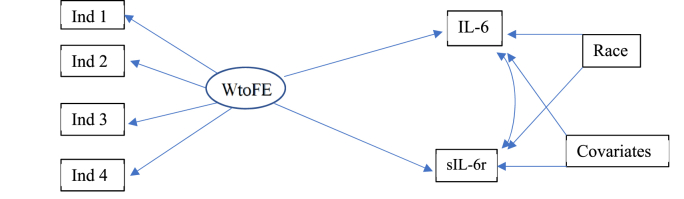
WtoFE and IL-6/sIL-6r Path-diagram.

Structural equation models (SEM) were utilized to evaluate inflammation as a latent factor of three major inflammatory biomarkers: IL-6, CRP, and fibrinogen, and also as a specific function of IL-6 and its soluble receptor (sIL-6r). The latent model has been utilized in prior studies of inflammation and chronic disease (Friedman et al., 2015; Marsland et al., 2010). As with previous research, we treated these markers initially as a single latent construct to assess the relatedness of the inflammatory cytokines (Fig. 1). We then breakdown this variable, isolating the IL-6/sIL-6r pathway (Fig. 2) in order investigate its role in the relationship between inflammation and work-family enrichment, as it has previously been indicated as the driver of the pro-inflammatory process.

### 3.5 Data analysis

The R packages Lavaan (Rosseel, 2012) and LavaanPlot were used to run multiple group structural equation modeling (SEM) analyses and to test measurement invariance. This approach was used to assess group differences between Whites and Blacks on WtoFE and FtoWE predicting inflammation. Three latent variables were constructed, one for WtoFE, one for FtoWE and one for inflammation. For each latent construct, the corresponding items served as manifest indicators. Model fit was assessed using fit indices: Comparative Fit Index (CFI), Standardized Root Mean Square Residual (SRMR) and Root Mean Square Error Approximation (RMSEA). Adequate fit is generally described as having a CFI greater than .9, SRMR less than 0.08 and RMSEA less than 0.06. Age, sex, education level, obesity, positive affect, extraversion, income perceived stress and co-morbidity of chronic disease were included as covariates. Preliminary analyses found stress and co-morbidity of chronic disease were not significantly associated with inflammation in the model and, thus, were removed from further analyses to conserve degrees of freedom and avoid oversaturation of the model.

*Note.* WtoFE = Work-to-family enrichment; IL-6 = serum interleukin-6 (pg/mL), sIL-6r = serum soluble interleukin-6 receptor (pg/mL).

## 4 Results

Characteristics of study participants are presented in Table 1. White individuals were, on average, older (48.75 years, *SD* = 12.61) than Black individuals (44.78 years, SD = 11.58). The Black sample had more female participants than males (65.85%), while the White sample had fewer females (45.73%) compared to males. Individuals in both groups were relatively highly educated, with 91.46% of Whites and 79.27% of Blacks having some college education. On average, Black participants had lower WtoFE, higher positive affect, higher extraversion, lower income and greater prevalence of obesity in comparison to Whites. However, FtoWE was similar between the two groups. There was no significant difference in the mean level of perceived stress between the two groups.

**Table 1:** Race-stratified Distribution of Participant Characteristics.

	White (n = 365)			Black (n = 82)			
Variable	N	%	Mean (SD)	N	%	Mean (SD)	*p*
**Age** (range 23–76)	363		48.75 (12.61)	82		44.78 (11.58)	0.0164
**Sex**							
Male	197	54.27		28	34.15		0.001
Female	166	45.73		54	65.85		
**Education**							
≤High School	31	8.54		17	20.73		0.001
≥ Some College	332	91.46		65	79.27		
**WtoFE**	362		11.17 (2.7)	80		10.25 (3.54)	0.0242
High (≥13)	119	32.78		18	21.95		
Average (10–12)	145	39.94		33	40.24		
Low (≤9)	99	27.27		31	37.81		
**FtoWE**	363		12.93 (2.87)	82		12.92 (2.97	0.6742
High (≥15)	118	32.5		24	29.3		
Average (12–14)	146	40.2		35	42.7		
Low (≤11)	99	27.27		23	28.0		
**Obesity (BMI** ≥ 30)	108	29.75		46	56.1		<0.001
**Comorbid chronic disease (>4)**	74	20.56		10	12.5		0.097
**Perceived stress scale**	342		22.11 (6.01)	80		23.8 (6.89)	0.057
**Positive Affect**	342		3.4 (0.71)	69		3.42 (0.81)	0.004
**Income**	342		$66,842.01 (50,466.03)	69		$41,132.35 (31,271.83)	<0.001
**Extraversion**	342		3.06 (0.6)	69		3.27 (0.56)	0.006224

*Note.* MIDUS: Midlife in the United States. WtoFE: Work-to-Family Enrichment, FtoWE: Family-to-Work Enrichment.

Non-transformed median levels of inflammatory biomarkers are shown for White MIDUS Refresher participants and Black MIDUS Refresher and Milwaukee Refresher participants in Table 2. Non-parametric Mann-Whitney *U* Test showed statistical differences between the two samples for all four biomarkers. Black participants had significantly lower median sIL-6r and higher IL-6, Fibrinogen and CRP concentrations. The inflammatory markers had non-normal distributions and were log transformed for subsequent multivariate analyses.

**Table 2:** Assay Range and Median Concentration of Participant Biomarker Values.

	White (n = 365)		Black (n = 82)		p
Biomarker	Assay Range	Median	Assay Range	Median	
Blood serum soluble interleukin-6 receptor (sIL-6r) (pg/mL)	10983.8–61276.6	32323.6	16498–49691.3	24140.6	<0.001
Blood serum interleukin-6 (IL-6) (pg/mL)	0.22–11.08	1.6	0.46–11.97	2.13	0.0026
Blood fibrinogen (mg/dL)	163–621	321.0	213–520	362.5	0.0002
Blood c-reactive protein (ug/mL)	0.07–79.3	1.05	0.09–13.56	2.06	0.0064

*Note*. MIDUS: Midlife in the United States.

**Fig. 3:**
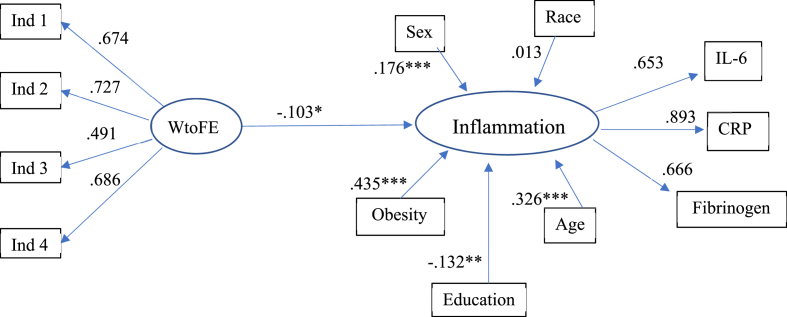
SEM Results of WtoFE predicting Inflammation, controlling for sex, education, age, obesity and race. *Note*. WtoFE = Work-to-family enrichment, RMSEA = 0.05, SRMR = 0.033, CFI = 0.96; *p < 0 0.05, **p < .01, ***p < .001.

Presented data are raw values prior to log transformation. Potential racial group differences in the relationship between WtoFE and inflammation were first assessed using a multiple group analysis. Prior to estimating the proposed model, we tested for measurement invariance in WtoFE and inflammation. The results indicated that configural measurement invariance held in WtoFE and inflammation. Table 3 shows standardized loadings of the latent variable constructs. Using multiple group analysis, the model with WtoFE and inflammation as latent variables and covariances between age and IL-6 and CRP was consistent with the data (χ^2^(92) = 154.658, *p* = .000, RMSEA = 0.057, CFI = 0.947, SRMR = 0.04). The results showed that magnitude of the estimated association of WtoFE with inflammation did not differ by race, while adjusting for sex, education, age, obesity, positive affect, income and extraversion (Δχ^2^(1) = 0.10899, *p* = .7413). Therefore, the White and Black groups were combined and a single-group SEM was estimated with race as an additional covariate. Positive affect, income and extraversion were not retained in the model due to non-significance and to improve model fit. This model was consistent with the data (χ^2^(31) = 75.727, *p* = .000, CFI = 0.96, RMSEA = 0.053, SRMR = 0.034). In this simplified model, WtoFE was significantly associated with inflammation (standardized estimate = -0.104, p = .031). The full model is indicated in Fig. 3. FtoWE was not significantly associated with inflammation for either racial group, after adjusting for covariates (results not shown).

**Table 3:** Standardized factor loadings of the latent constructs.

Latent Construct	Indicator	White (n = 365)	Black (n = 82)
Inflammation	Blood c-reactive protein	0.925	0.814
	Blood serum interleukin-6 (IL-6)	0.674	0.532
	Blood fibrinogen/100	0.637	0.763
WtoFE	Indicator 1	0.687	0.574
	Indicator 2	0.688	0.714
	Indicator 3	0.410	0.698
	Indicator 4	0.634	0.862

*Note*. WtoFE = Work-to-family enrichment. Model fit indices: RSMEA = 0.065, SRMR = 0.043, CFI = 0.941.

**Fig. 4:**
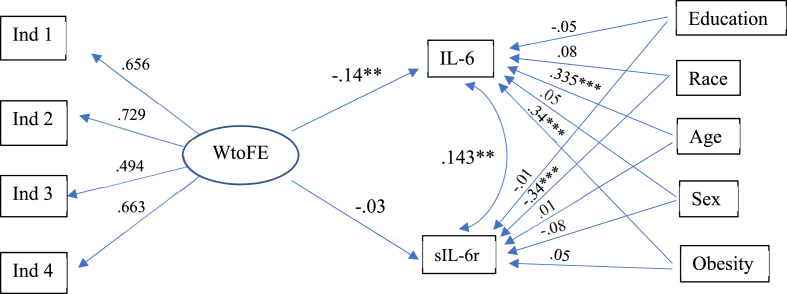
Summary of the results from a path diagram of WtoFE and IL-6 and sIL-6r, controlling for sex, education, age, obesity and race. *Note*. WtoFE = Work-to-family enrichment, IL-6 = Interleukin-6, sIL-6r = interleukin-6 soluble receptor; CFI = 0.972, RMSEA = 0.044, SRMR = 0.029; *p < .05, **p < .01, ***p < .001.

Finally, we estimated a multiple group model with paths between WtoFE and two non-independent indicators of inflammation, IL-6 and sIL-6r. IL-6 and sIL-6r were positively correlated with one another (0.10) indicating a relationship between the two biomarkers. The fit of this model accounts for covariance between IL-6 and sIL-6r and that includes all the covariates was good (χ^2^(37) = 58.774, CFI = 0.962, RMSEA = 0.051, SRMR = 0.03). However, the results from the multigroup model indicated that the estimated association of WtoFE with IL-6 and sIL-6r did not differ by race, while adjusting for covariates (Δχ^2^(1) = 1.4627, *p* = .2265). Therefore, the final model was a single-group SEM was estimated with race as an additional covariate (Fig. 4). The results showed WtoFE predicted lower IL-6 (−0.14, p = .003) but not sIL-6r (−0.03, p = .579).

## 5 Discussion

The present study is one of the first to exhibit an association between a synergistic work and family life and measures of inflammation. In a national sample of 447 adults in the US, higher levels of WtoFE was associated with less systemic inflammation as well as lower concentrations of serum IL-6. However, it should be noted that significance was not found between FtoWE and systemic inflammation nor IL-6. A recent MIDUS study, building from Greenhaus and Powell's findings (Greenhaus and Powell, 2006), explained that over time, work-family enrichment may generate a positive arsenal of resources, resulting in a reduced susceptibility to stressors and in turn better inflammatory profiles (Russo, 2015). Our findings move a step further by showing the resources gained at work may have a more substantial effect on inflammatory mediated health processes than the countercurrent from family. Lee, Chang, and Kim suggested similarly that FtoWE may not be as beneficial, since family situations often become an additional source of stress for people, depleting their resources rather than multiplying them (Lee et al., 2011).

Work-family enrichment continues to display potential clinical and public health value (Carlson et al., 2010; Gareis et al., 2009; Russo, 2015; Schnettler et al., 2022; Versey and Tan, 2020). While a growing body of research has linked positive experiences to lower inflammation (Chiang et al., 2015; Ong et al., 2018; N. L. Sin et al., 2015), there is a dearth of literature that explores this relationship among the work-family interface; as the predominant focus in inflammatory studies remains on stressors and stressor exposure (Diamond et al., 2021; Rohleder, 2019; Tondokoro et al., 2021). Furthermore, most previous studies on positivity and inflammation have investigated shear number of experiences or personality traits as an explanation of correlation (Ironson et al., 2018; Schenk et al., 2018; N. Sin and Almeida, 2018). We addressed both shortcomings by evaluating the perceived benefit of work-family enrichment while controlling for potentially confounding variables such as trait disposition and recent positive affect. In doing so, we demonstrated that WtoFE, in itself, may be a positive experience that is uniquely associated to lower levels of systemic inflammation and IL-6. Covariate adjustment for personality (i.e., extraversion) and positive affect did not attenuate the WtoFE effect. These results suggest that work-family enrichment is not simply an optimistic worldview as suggested by (Kim et al., 2021). Instead, it seems that work-family enrichment is a positive experience that, similarly to others (Bono et al., 2013; N. L. Sin et al., 2015), suggests that favorable events and circumstances, like a well-fitted work-family arrangement, may impede pro-inflammatory processes and presumably decrease risk of CVD.

Our study is further innovative in examining sIL-6r, as no previous study to our knowledge has examined this pro-inflammatory marker in the positivity literature. It is noteworthy that we found no association of work family enrichment with sIL-6r, in part because this marker holds clinical promise as an inflammatory target in combating CVD (Casas and Hingorani, 2012). Nonetheless, there may be an indirect effect of WtoFE for sIL-6r because IL-6 and sIL-6r have a small positive correlation in our study, and WtoFE is clearly associated with IL-6. We did not test this possibility, but it along with ratio indicators of sIL-6r:IL-6 may be worthy of further research. A recent study consisting of myocardial infarction patients showed that higher IL-6 levels and a lower sIL-6r:IL-6 ratio early after presentation was associated with larger infarct size and decreased cardiovascular function (Groot et al., 2019). If these observations are meaningful in the general population, the isolated decrease in IL-6 among those with higher WtoFE in our study may provide preliminary insight into possible cardiovascular benefits of work-family enrichment.

The focus on inflammation in the current study was motivated by the well-documented link with cardiovascular health disparities seen among Blacks (Furman et al., 2019; Libby and Hansson, 2015; Shrivastava et al., 2015). Blacks in our study had higher levels of pro-inflammatory markers at baseline. While elevated levels of proinflammatory markers may place Blacks at a disproportionally higher risk of myocardial infarction and decreased cardiovascular function compared to their White counterparts (Libby and Hansson, 2015), we cannot eliminate work-family enrichment (both WtoFE and FtoWE) as a possible source of racial variation in CVD. Our data also indicates that Blacks experience less WtoFE than Whites. Given that it is associated with inflammation, WtoFE may mediate racial differences in inflammatory markers, in turn altering CVD risk. However, this is not reflected by the model used in the current study, so a definitive conclusion cannot be drawn.

## 6 Limitations

Our multi-group models concluded no difference by race in the association of WtoFE with inflammation (systemic inflammation or the IL-6-sIL-6r pathway). However, the size of the Black group is small, raising questions of power (the sufficiency of the sample to detect meaningful differences between Whites and Blacks), which was exaggerated by the level of covariate adjustment needed to isolate the possible effects of work-family enrichment from positive affectivity and personality-related confounds. Furthermore, the available data was cross-sectional, which does not allow for the assessment of changes in inflammation over time. We also cannot eliminate the possibility that IL-6 could drive both sets of results because it was in the dependent variable for both models.

## 7 Future direction

Future research should focus on identifying distinct aspects of work that benefit family to better understand how WtoFE exerts its effect on inflammation. This may be done by examining the correlation between job characteristics (ie. type, hours, field) and work environment on varying family structures. Studies should also examine the relationship between WtoFE and other clinical risk factor profiles (i.e., smoking, high cholesterol), along with other inflammatory markers which may help identify its overall effect on cardiometabolic diseases. Although sustained inflammation has been shown to aggravate progression of CVD, research has not clearly defined why these processes are heightened among Black Americans. Continued efforts in the recruitment and retention of Blacks in studies are warranted – as data on inflammatory biomarkers among Blacks in the U.S. are limited by the small numbers of Black subjects enrolled in epidemiologic cohorts.

## 8 Conclusion

This study advances the current literature by establishing a correlation between WtoFE and inflammation and provides an additional explanation for the associations between work-family enrichment and positive health outcomes. Assessing and intervening on imbalances among one's work-family life may serve as a powerful starting point to both prevent and reduce negative effects of inflammation on health. Although differences between racial groups remain undetermined, increasing WtoFE may be a promising method of attenuating chronic illnesses such as CVD which disproportionally affects Black Americans.

## Funding

This research was supported by a grant from the 10.13039/100000049National Institute on Aging (1U19AG051426).

## Declaration of competing interest

The authors declare that they have no known competing financial interests or personal relationships that could have appeared to influence the work reported in this paper.

## Data Availability

Data will be made available on request.
