# Supplementary material for: Work-family enrichment: A potential buffer of inflammation among black adults?
Source: Brain Behav Immun Health. 2022 Sep 19;25:100517. doi: 10.1016/j.bbih.2022.100517 (PMC9508348; doi:10.1016/j.bbih.2022.100517)
Supplement: Multimedia component 1 [file mmc1.docx]

**Supplementary Material:**

**Table S1:** *Results of the test of measurement invariance*

| Measurement invariance | RMSEA | CFI | SRMR | AIC | BIC | *df* | χ^2^ | ∆χ^2^ | ∆*df* | *p* |
| --- | --- | --- | --- | --- | --- | --- | --- | --- | --- | --- |
| **Inflammation** |  |  |  |  |  |  |  |  |  |  |
| Configural | .000 | 1.000 | .000 | 2683.5 | 2766.6 | 0 | 0.000 |  |  |  |
| Weak | .076 | .992 | .027 | 2685.8 | 2759.7 | 2 | 6.346 | 6.346 | 2 | .042 |
| Strong | .073 | .986 | .034 | 2687.5 | 2752.1 | 4 | 12.027 | 5.682 | 2 | .058 |
| Strict | .058 | .984 | .048 | 2685.4 | 2736.2 | 7 | 15.899 | 3.871 | 3 | .276 |
| **Work-family enrichment** |  |  |  |  |  |  |  |  |  |  |
| Configural | .126 | .958 | .027 | 16347 | 16476 | 4 | 53.286 |  |  |  |
| Weak | .093 | .960 | .027 | 16342 | 16454 | 7 | 53.978 | .710 | 3 | .871 |
| Strong | .089 | .948 | .032 | 16354 | 16450 | 10 | 71.993 | 18.015 | 3 | <.001 |
| Strict | .089 | .928 | .042 | 16374 | 16449 | 14 | 99.843 | 27.851 | 4 | <.001 |

*Note*. RMSEA = Root Mean Square Error of Approximation, CFI = Comparative Fit Index, SRMR = Standardized Root Mean Squared Residual, AIC = Akaike Information Criterion, BIC= Bayesian Information Criterion, *df* = degrees of freedom.
